# Detecting false positive sequence homology: a machine learning approach

**DOI:** 10.1186/s12859-016-0955-3

**Published:** 2016-02-24

**Authors:** M. Stanley Fujimoto, Anton Suvorov, Nicholas O. Jensen, Mark J. Clement, Seth M. Bybee

**Affiliations:** Computer Science Department, Brigham Young University, Provo, Utah 84602 USA; Department of Biology, Brigham Young University, Provo, Utah 84602 USA

**Keywords:** Homology, Orthology, Paralogy, Machine learning, Evolution, RNA-seq

## Abstract

**Background:**

Accurate detection of homologous relationships of biological sequences (DNA or amino acid) amongst organisms is an important and often difficult task that is essential to various evolutionary studies, ranging from building phylogenies to predicting functional gene annotations. There are many existing heuristic tools, most commonly based on bidirectional BLAST searches that are used to identify homologous genes and combine them into two fundamentally distinct classes: orthologs and paralogs. Due to only using heuristic filtering based on significance score cutoffs and having no cluster post-processing tools available, these methods can often produce multiple clusters constituting unrelated (non-homologous) sequences. Therefore sequencing data extracted from incomplete genome/transcriptome assemblies originated from low coverage sequencing or produced by *de novo* processes without a reference genome are susceptible to high false positive rates of homology detection.

**Results:**

In this paper we develop biologically informative features that can be extracted from multiple sequence alignments of putative homologous genes (orthologs and paralogs) and further utilized in context of guided experimentation to verify false positive outcomes. We demonstrate that our machine learning method trained on both known homology clusters obtained from OrthoDB and randomly generated sequence alignments (non-homologs), successfully determines apparent false positives inferred by heuristic algorithms especially among proteomes recovered from low-coverage RNA-seq data. Almost ~42 % and ~25 % of predicted putative homologies by InParanoid and HaMStR respectively were classified as false positives on experimental data set.

**Conclusions:**

Our process increases the quality of output from other clustering algorithms by providing a novel post-processing method that is both fast and efficient at removing low quality clusters of putative homologous genes recovered by heuristic-based approaches.

**Electronic supplementary material:**

The online version of this article (doi:10.1186/s12859-016-0955-3) contains supplementary material, which is available to authorized users.

## Background

One of the most fundamental questions of modern comparative evolutionary phylogenomics is to identify common (homologous) genes that originated through complex biological mechanisms such as speciation, multiple gene losses/gains, horizontal gene transfers, deep coalescence, etc. [1]. When homologous sequences are identified, they are usually grouped and aligned together to form clusters. Homologous DNA (and those translated to amino acids) sequences can be further subdivided into two major classes: orthologs and paralogs. Orthologs are defined as homologous genes in different species that arose due to speciation events, whereas paralogs have evolved from gene duplications. Moreover, orthologous genes are more likely to exhibit a similar tempo and mode of evolution, thus preserving overall sequence composition and physiological function. Paralogs, instead, tend to follow different evolutionary trajectories leading to subfunctionalization, neofunctionalization or both [2]. Nevertheless this phenomenon, called the ortholog conjecture, is still debatable [3] and requires additional validation since it has been shown that even between closely related species some orthologs can diverge such that they eventually loose common functionality.

The accurate detection of sequence homology and subsequent binning into aforementioned classes is essential for robust reconstruction of evolutionary histories in the form of phylogenetic trees [4]. To date, numerous computational algorithms and statistical methods have been developed to perform orthology/paralogy assignments for genic sequences (for review see [5]). Methodologically these approaches employ heuristic-based or evidence (phylogenetic tree)-based identification strategies, which produces varying frequencies of false positive or negative results. The majority of heuristic algorithms rely on the principle of Reciprocal Best Hit (RBH, [6]) where BLAST [7] hit scores (e-values) approximate evolutionary similarity between two biological sequences. Further algorithmic augmentations of those heuristics, for instance Markov graph clustering (unsupervised learning) [8], enables the definition of orthologous/paralogous clusters from multiple pairwise comparisons. Despite their relatively low computational complexity, these algorithms have been shown to overestimate the number of putative homologies (i.e., higher rates of false positive detection compared to evidence-based methods [9]).

In this current era of next-generation sequence data researchers have gained access to tremendous amounts of “omic” data, including for non-model organisms. Phylogenetic information, including species trees, is very limited, unreliable and/or completely unavailable for some poorly studied taxa, thus evidence-based methods are not directly applicable to infer homology. Ebersberger et al. [10] developed the first attempt to circumvent this problem, using a novel hybrid approach (HaMStR) for extraction of homologous sequences from EST/RNA-seq data using a profile Hidden Markov Model (pHMM) [11] based on a similarity search coupled with subsequent RBH derived from re-BLASTing against a reference proteome. The innovative feature of their approach is in the utilization of pHMM as an additional evidence for homology. This architecture incorporates characteristics of multiple sequence alignments (MSA) for user pre-defined core orthologs. Then, a HMM search is performed with each individual pHMM using matching criterion applied to find putative orthologs in the proteome of interest. This method, however, has limitations and weaknesses, such as

i) Proteome training sets composed of phylogeneticaly “meaningful” taxa for construction of core ortholog clusters may not be available,
ii) Identification of informative core ortholog clusters may be somewhat cumbersome due to incomplete and/or low coverage sequencing,
iii) The pHMMs may not contain any relevant compositional or phylogenetic properties about biological sequences that constitute MSA, and
iv) Inability to explicitly identify paralogy limits the use of HaMStR for some evolutionary applications. Hence, homologous clusters inferred from various multiple sequences require further validation to improve confidence in orthology/paralogy classification.

Here, we propose a unique approach to identify false positive homologies detected by heuristic methods, for example HaMStR or InParanoid [12]. Our machine learning method uses phylogenetically-guided inferred homologies to identify non-homologous (false positive) clusters of sequences. This improves the accuracy of heuristic searches, like those that rely on BLAST.

## Methods

### Library preparation and RNA-seq

For the experimental data set (OD_S) we used 18 Odonata (dragonflies and damselflies) and 2 Ephemeroptera (mayflies) species. Total RNA was extracted from the eye tissues of each taxon using NucleoSpin RNA II columns (Clontech) and reverse-transcribed into cDNA libraries using the Illumina TruSeq RNA v2 sample preparation kit that both generates and amplifies full-length cDNAs. Prepped Ephemeroptera mRNA libraries were sequenced on an Illumina HiSeq 2000 producing 101 bp paired-end reads by the Microarray and Genomic Analysis Core Facility at the Huntsman Cancer Institute at the University of Utah, Salt Lake City, UT, USA, while all Odonata preps were sequenced on a GAIIx producing 72 bp paired-end reads by the DNA sequencing center at Brigham Young University, Provo, UT, USA. The expected insert sizes were 150 bp and 280 bp respectively. Raw RNA-seq reads were deposited in the National Center for Biotechnology Information (NCBI), Sequence Read Archive, see Additional file 1.

### Read trimming and *de novo* transcriptome assembly

The read libraries were trimmed using the Mott algorithm implemented in PoPoolation [13] with default parameters (minimum read length = 40, quality threshold = 20). For the assembly of the transcriptome contigs we used Trinity [14], currently the most accurate *de novo* assembler for RNA-seq data [15], under the default parameters.

### Downstream transcriptome processing

In order to identify putative protein sequences within the Trinity assemblies we used TransDecoder (http://transdecoder.github.io), the utility integrated into the comprehensive Trinotate pipeline (http://trinotate.github.io) that is specifically developed for automatic functional annotation of transcriptomes [16]. TransDecoder identifies the longest open reading frames (ORFs) within each assembled DNA contig, the subset of the longest ORFs is then used to empirically estimate parameters for a Markov model based on hexamer distribution. The reference null distribution that represents non-coding sequences is constructed by randomizing the composition of these longest contigs. During the next decision step, each longest determined ORF and its 5 other alternative reading frames are tested using the trained Markov model. If the log-likelihood coding/noncoding ratio is positive and is the highest, this putative ORF with the correct reading frame is retained in the protein collection (proteome). For more details about the RNA-seq libraries, assemblies and predicted proteomes see Additional file 1.

### Construction of *Drosophila* data set

Ten high quality *Drosophila* raw RNA-seq data sets (DROSO) were obtained from NCBI (Additional file 2). First we trimmed the reads using PoPoolation [13] and subsampled the read libraries to the size of the smallest (*Drosophila biarmipes*). Then, two additional data sets corresponding to 50 % and 10 % of the scaled libraries were constructed by randomly drawing reads from the original full-sized libraries. Finally, *de novo* transcriptome assembly and protein prediction were conducted as outlined above for these three data sets. These data sets were used to test whether homology clusters derived from low-coverage RNA-seq libraries contain more false positives.

### Gene homology inference

To predict probable homology relationships between proteomes we used the heuristic predictor InParanoid/MultiParanoid based on the RBH concept [12, 17]. Among various heuristic-based methods for sequence homology detection, OrthoMCL [8] and InParanoid [12] have been shown to exhibit comparable high specificity and sensitivity scores estimated by Latent Class Analysis [9], so in the present study we exploited InParanoid/MultiParanoid v. 4.1 for the purpose of simplicity in computational implementation. InParanoid initially performs bidirectional BLAST hits (BBHs) between two proteomes to detect BBHs in the pairwise manner. For this step, we set default parameters with the BLOSUM62 protein substitution matrix and bit score cutoff of 40 for all-against-all BLAST search. Next, MultiParanoid forms multi-species groups using the notion of a single-linkage. Due to inefficient MultiParanoid clustering algorithm, we had to perform a transitive closure to compile homology clusters for all species together. Transitive closure is an operation performed on a set of related values. Formally, a set S is transitive if the following condition is true: for all values A, B, and C in S, if A is related to B and B is related to C, then A is related to C. Transitive closure takes a set (transitive or non-transitive) and creates all transitive relationships, if they do not already exist. When a set is already transitive, its transitive closure is identical to itself. In the case of the pairwise relationships produced by InParanoid, we constructed orthologous clusters using the notion of transitive closure, where gene identifiers were the values, and homology was the relationship.

For example, our OD_S data set consisted of *N* = 20 proteomes, so we had to perform N×(N - 1)/2 = 190 pairwise InParanoid queries. A simple transitive closure yielded total 13,998 homology clusters for OD_S. The DROSO data set yielded 20,676, 18,584 and 17,067 homology clusters for 100 %, 50 % and 10 % respectively. Then putative homologous genes were aligned to form individual MSA homology clusters for the subsequent analyses using MAFFT v. 6.864b [18] with the “-auto” flag that enabled detection of the best alignment strategy between accuracy- and speed-oriented methods.

Additionally, we utilized HaMStR v. 13.2.3 [10] under default parameters to delineate putative orthologous sequences in the OD_S proteome sets. 5,332 core 1-to-1ortholog clusters of 5 arthropod species (*Ixodes scapularis, Daphnia pulex, Rhodnius prolixus, Apis mellifera* and *Heliconius melpomene*) for training pHMM were retrieved from the latest version of OrthoDB [19]. We used *Rhodnius prolixus* (triatomid bug) as the reference core proteome because this is the closest phylogenetically related species and publically available proteome to the Ephemeroptera/Odonata lineage [20]. As previously described, each core ortholog cluster was aligned to create MSA using MAFFT and converted into HMM profile using HMMER v. 3.0 [21]. BBHs against the reference proteome were derived using reciprocal BLAST.

### Construction of ground-truth training sets

The OrthoDB database is one of the most comprehensive collections of putative orthologous relationships predicted from proteomes across a vast taxonomic range [19]. This data is particularly useful for construction of training sets since OrthoDB clusters were detected using a phylogeny-informed approach collated with available functional annotations. Hence, training sets constructed from OrthoDB clusters have the inherent benefit of both an evolutionary and physiological assessment resulting in more precise filtering for false positive homology.

The key to our method was the development of labeled training sets that were used to train supervised machine learning classifiers. Previously, homology clusters were known and annotated in OrthoDB. There were, however, no annotated clusters that represented non-homology clusters from random alignments. Thus, we created and annotated our own set of non-homology clusters through a generative process. We created these clusters in two different manners: randomly aligned sequences and evolving sequences from the homology clusters.

We extracted 5,332 homology (H) clusters from the predefined OrthoDB profile called “single copy in > 70 % of species” across the entire arthropod phylogeny in the database, and then aligned them. Non-homology (NH) clusters were generated using: i) the alignment of randomly drawn sequences from the totality of the protein sequences with cluster size sampled from Poisson (λ), where λ = 44.3056 was estimated as the average cluster size of Hs and ii) by evolving the sequences taken from H clusters. This process of evolving sequences was accomplished by using PAML [22] to generate random binary trees for each sequence within a cluster. The discretized number of terminal branches for each random tree was sampled from a normal distribution with mean 50 and a standard deviation of 15. Within each of the clusters, individual sequences were evolved using their respective randomly generated tree using Seq-Gen [23]. We used WAG + I [24] as the substitution model for the amino acid sequences during the evolving process specifying the number of invariable sites (−i) at 0 %, 25 % and 50 %. Then, to form NH clusters, a single evolved sequence from the terminal branches was selected randomly from each tree. By doing so, we simulated more realistic clusters in which the evolved sequences were diverged enough to be considered as non-homologous to each other.

From the H and NH clusters, two different sets of training, validation and testing partitions were formed. The first set (EQUAL) had an equal number of homology, randomly aligned, 0 % invariable-site evolved, 25 % invariable-site evolved and 50 % invariable-site evolved clusters within the combination of training, validation and testing data sets. The second set (PROP) consisted of 50 % of the training set as homology clusters while the remaining half of the training set was composed of equal parts randomly aligned, 0 % invariable-site evolved, 25 % invariable-site evolved and 50 % invariable-site evolved clusters. The combined data sets were then partitioned into training, validation and testing. This was done by randomly sampling from the pool of clusters and assigning 80 % of the clusters (8,800) to training, 10 % (1,100) to validation and the last 10 % (1,100) to testing.

### Attribute selection

Ten different attribute features were selected (Table 1) and calculated for individual MSA of putative homology clusters and for training Hs and NHs as well. To identify randomly aligned positions in MSAs, we utilized ALISCORE [25], software based on the principle of parametric Monte Carlo resampling within a sliding window. This approach is more objective and exhibits less conservative behavior contrasted to commonly used non-parametric approaches implemented in GBLOCKS [26, 27]. We expected the number of randomly aligned positions for false positive homologies to be higher than for true homologs. Additionally, several other simple metrics (the number of sequences forming MSAs, alignment length, total number of gaps, total number of amino acid residues and range defined as the difference between longest and smallest sequences within MSAs) were also derived. Overall, incorporation of these attributes into a training set was used to increase the robustness of the performance of the machine learning algorithm. We also obtained amino acid composition for each sequence from each cluster and binned it into four classes according to physicochemical properties of amino acids (charged, uncharged, hydrophobic and special cases), then compositional dispersion was calculated using an unbiased variance estimator corrected for sequence length. Here we assumed that amino acid composition between closely related sequences would be preserved by analogous weak genome-wide evolutionary constraints [28, 29] and thus have diminished variance.

**Table 1:** All Features that were used in order to train the machine learning algorithm. Each of these features was calculated for each of the clusters

Feature	Description
Aliscore	The number of positions identified by Aliscore as randomly aligned
Length	The length of the alignment
# of Sequences	The number of sequences in the alignment
# of Gaps	Number of base positions marked with a gap
# of Amino Acids	Number of amino acids in the alignment
Range	Longest non-aligned sequence length minus shortest non-aligned sequence length
Amino Acid Charged	Standard deviation for the proportions of amino acids in the charged class for each sequence
Amino Acid Uncharged	Standard deviation for the proportions of amino acids in the uncharged class for each sequence
Amino Acid Special	Standard deviation for the proportions of amino acids in the non-charged and non-hydrophobic class for each sequence
Amino Acid Hydrophobic	Standard deviation for the proportions of amino acids in the hydrophobic class for each sequence

### Machine learning

For detection of false positive homology we utilized different supervised machine learning algorithms in order to learn from the labeled data instances. Supervised machine learning algorithms take in labeled instances of a particular event as input. From these labeled instances, the algorithm can then learn from the features associated with the instance to perform classification on other, unlabeled instances. A number of different algorithms were used in order to find a model that performed well. Waikato Environment for Knowledge Analysis (WEKA) software [30] was utilized for training different supervised machine learning classifiers and for evaluating the test data sets. A set of models was trained and compared using the arthropod data set (see Training data sets for additional information).

A number of different machine learning algorithms were evaluated. These algorithms included: neural networks, support vector machines (SVMs), random forest, Naive Bayes, logistic regression, and two meta-classifiers. A total of seven models were trained for the arthropod data set. A meta-classifier uses a combination of machine learning algorithms in tandem to perform classification. The two different meta-classifiers utilized stacking with a neural network as the meta-classifying algorithm. Stacking takes the output classifications for all other machine learning algorithms as input and then feeds them into another machine learning algorithm. The learning algorithm that is stacked on the others is then trained and learns which machine learning algorithms it should give more credence when performing classification. One of the meta-classifiers incorporated all the previously mentioned learning algorithms (neural network, SVM, random forest, Naive Bayes, and logistic regression). The other meta-classifier used all the previously mentioned learning algorithms except for logistic regression. All parameters for each machine learning algorithm are summarized in Table 2.

**Table 2:** The machine learning parameters used for each of the different algorithms in WEKA

Algorithm	Parameters
Neural Network	weka.classifiers.functions.MultilayerPerceptron -L 0.1 -M 0.05 -N 3000 -V 0 -S 0 -E 40 -H a
Support Vector Machine (SVM)	weka.classifiers.functions.SMO -C 1.0 -L 0.001 -P 1.0E-12 -N 0 -V −1 -W 1 -K “weka.classifiers.functions.supportVector.PolyKernel -C
Random Forest	weka.classifiers.trees.RandomForest -I 10 -K 0 -S 1
Naive Bayes	weka.classifiers.bayes.NaiveBayes
Logistic Regression	weka.classifiers.functions.Logistic -R 1.0E-8 -M −1
Meta-Classifier w/o Logistic Regression	weka.classifiers.meta.Stacking -X 10 -M “weka.classifiers.functions.MultilayerPerceptron -L 0.3 -M 0.2 -N 500 -V 0 -S 0 -E 20 -H a” -S 1 -B “weka.classifiers.trees.RandomForest -I 10 -K 0 -S 1” -B “weka.classifiers.bayes.NaiveBayes ” -B “weka.classifiers.functions.SMO -C 1.0 -L 0.001 -P 1.0E-12 -N 0 -V −1 -W 1 -K “weka.classifiers.functions.supportVector.PolyKernel -C 250007 -E 1.0””
Meta-Classifier w/Logistic Regression	weka.classifiers.meta.Stacking -X 10 -M “weka.classifiers.functions.MultilayerPerceptron -L 0.3 -M 0.2 -N 500 -V 0 -S 0 -E 20 -H a“ -S 1 -B ”weka.classifiers.functions.Logistic -R 1.0E-8 -M −1” -B “weka.classifiers.functions.MultilayerPerceptron -L 0.3 -M 0.2 -N 500 -V 0 -S 0 -E 20 -H a” -B “weka.classifiers.trees.RandomForest -I 10 -K 0 -S 1” -B “weka.classifiers.bayes.NaiveBayes ” -B “weka.classifiers.functions.SMO -C 1.0 -L 0.001 -P 1.0E-12 -N 0 -V −1 -W 1 -K “weka.classifiers.functions.supportVector.PolyKernel -C 250007 -E 1.0””

### Training

The training data set was used as input to the machine learning model for parameter selection. For the arthropod data set, 80 % of the data were used for training, while 10 % of the data was reserved for validation and the last 10 % for testing. Machine learning algorithms were utilized to learn from the combination of the H and NH clusters in the data set to differentiate the two. A trained model could then be used to classify unlabeled instances as homologous and non-homologous. There were a total of 8,800 instances in the OrthoDB arthropod data set that were used as a training set for both the PROP and the EQUAL data sets. In the PROP data set, there were 4,378 H and 4,422 NH clusters. In the EQUAL data set, there were 1,753 H and 7,047 NH clusters.

### Validation

The validation data sets were used after the model had been trained on the training data set. By using the trained model on the validation set, the efficacy of the model could be seen. 10 % of the arthropod data set formed the arthropod validation set. The models trained using the arthropod training set were validated only with the arthropod instances. If the model did not perform adequately on the validation set, different parameters for the machine learning algorithms were modified in an attempt to improve the performance of the models. The re-trained models would then revalidate on their same, respective validation sets. The process was repeated until adequate performance of the learning algorithm was reached. The OrthoDB arthropod validation set consisted of 1,100 instances for both the PROP and EQUAL data sets. The PROP data set had 566 H and 534 NH clusters. The EQUAL data set had 238 H and 862 NH clusters.

### Testing

**Fig. 1:**
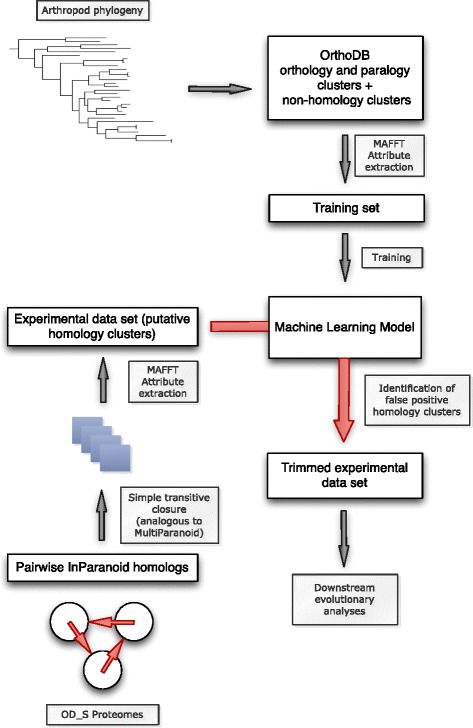
A diagram of the workflow. This figure shows the different steps that were used in developing our machine learning model. Arthropod phylogeny was generated in previous studies and deposited in OrthoDB. These sequences were then gathered from OrthoDB and used as our orthology and paralogy clusters. They were combined with generated non-homology clusters. The combination represents our training data set used to train the machine learning algorithms. The experimental data were assembled with proteins inferred from the assemblies. InParanoid was then used to identify putative homologs. Once putative homologs were identified they were input into the trained machine learning algorithms for classification and subsequent cluster trimming

All general steps of our pipeline are summarized in Fig. 1 using the example of OD_S processing. Testing data sets were used only after all the models were finished being trained and validated. This is to ensure an honest measure of the predictive capacity of the models because the testing data were never used in order to evaluate how our model was built and to modify the models. The last 10 % of the arthropod data set was used as the arthropod test set. The arthropod test set from the OrthoDB contained 1,100 instances for both the PROP and Equal data sets. The PROP data set had 555 H and 545 NH clusters. The EQUAL data set had 207 H and 893 NH clusters.

### Performance evaluation

We tested our filtering process by applying the arthropod classifiers trained on the ground-truth data set to the DROSO and OD_S data sets. Unlike the testing sets mentioned in the previous section, the ground-truth for these data sets was unknown. We examined the number of clusters filtered and conducted a manual inspection of a subset of the filtered clusters to verify the removal of only false positive homology clusters. Because there are, to the authors’ knowledge, no other post-processing methods for cluster filtering that exist our approach is novel. The filtering processes that do exist are heuristic-based approaches, such as an e-value cutoff, that are built-in modules of the clustering software. Therefore, for comparison, we only examined the number of clusters filtered from the output of InParanoid and HaMStR.

## Results and discussion

**Fig. 2:**
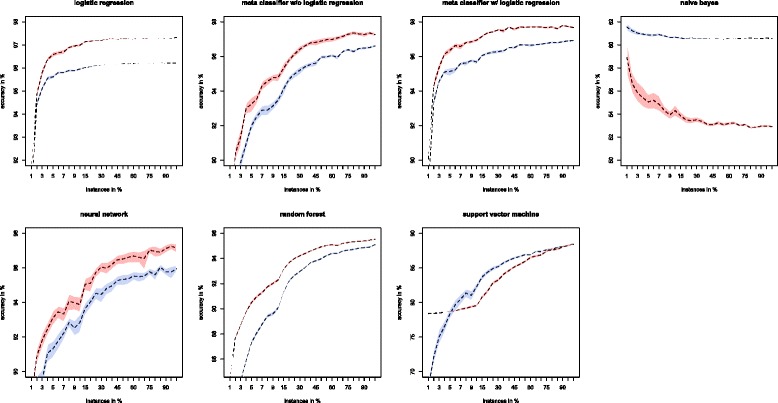
Bootstrapping results for the machine learning models. Bootstrapping was conducted using 100 replicates for each classifier. Error envelopes can also be seen for each classifier. Except for Naive Bayes, as the percentage of total training instances used during learning increases accuracy increases and the error envelope decreases

**Fig. 3:**
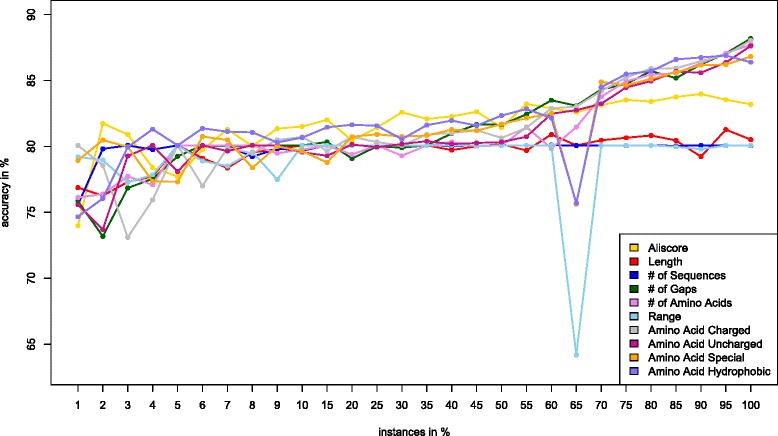
Accuracy curves for individual features (EQUAL training data set) using meta-classifier w/ logistic regression. The number of gaps, amino acid composition and number of amino acids features exhibit better predictive accuracy

As can be seen in Table 3 for both the PROP and EQUAL data sets, the arthropod models all (with the exception of Naive Bayes and SVM) had classification accuracies higher than 96 % on the validation set. On the test set, all models (with the exception of Naive Bayes and SVM) had classification accuracies higher than 95 %. The algorithms that performed the strongest were the meta-classifiers. The meta-classifier using logistic regression performed best in both the PROP and EQUAL data sets. Comparing the two different data sets, the models perform similarly whether given the PROP or EQUAL data sets. The only exception to this is the Naive Bayes classifier that performs much better (~8 % accuracy increase) when given the PROP data set. However, the models trained with the EQUAL data sets were slightly better in terms of accuracy (Fig. 2). In the arthropod models, we varied the size of the training set (from 1 % to 100 % of training instances). The validation set accuracy of the meta-classifier with logistic regression plateaued and slowed growth after training on 5 % or more of the training instances. Before this, their classification accuracies of all models were erratic with both increases and decreases as the training set size increased. The models behave differently when given varied amounts of data to train on (Fig. 2). All models except for Naive Bayes increased in accuracy as the training data grew. Logistic regression and the meta-classifier with logistic regression required the least amount of training data before they started to plateau. Additionally we tested which features were the most meaningful for classification using meta-classifier with logistic regression (Fig. 3). The “number of gaps” feature provided the best accuracy when 100 % of instances were used. Since increased indel events are accumulated over longer evolutionary time periods, the inferred MSAs from such highly diverged sequences with lost signatures of common ancestry are expected to have multiple gaps. Moreover, clusters prone to large amounts of missing data will be classified as NH using this feature. Similar accuracy levels were achieved for the four amino acid composition and number of amino acids features. As we mentioned earlier, selection forces may preserve amino acid composition especially through the action of purifying selection [31] making these features useful for H vs. NH cluster discrimination. Other features, except for Aliscore that exhibited an intermediate accuracy, had accuracy < 80 %, which might be explained by the fact that these features are less biologically meaningful.

**Table 3:** Summary of arthropod machine learning model performance

	OrthoDB Arthropod EQUAL		OrthoDB Arthropod PROP	
Algorithm	Validation	Testing	Validation	Testing
Neural Network	97.1815 %	96.8153 %	97.5452 %	96.5423 %
Suppor Vector Machine (SVM)	89.1351 %	88.0801 %	88.0668 %	88.2621 %
Random Forest	98.1362 %	95.9054 %	97.8748 %	95.5414 %
Naive Bayes	53.0628 %	52.5023 %	61.2229 %	60.3276 %
Logistic Regression	96.5905 %	97.2702 %	96.3064 %	96.3603 %
Meta-Classifier w/o Logistic Regression	98.5112 %	98.3621 %	98.5907 %	96.8153 %
Meta-Classifier w/ Logistic Regression	98.6362 %	97.7252 %	98.5680 %	97.5432 %

This table shows the performance of each of the different learning algorithms that were trained, validated, and tested with the OrthoDB arthropod gene clusters

**Fig. 4:**
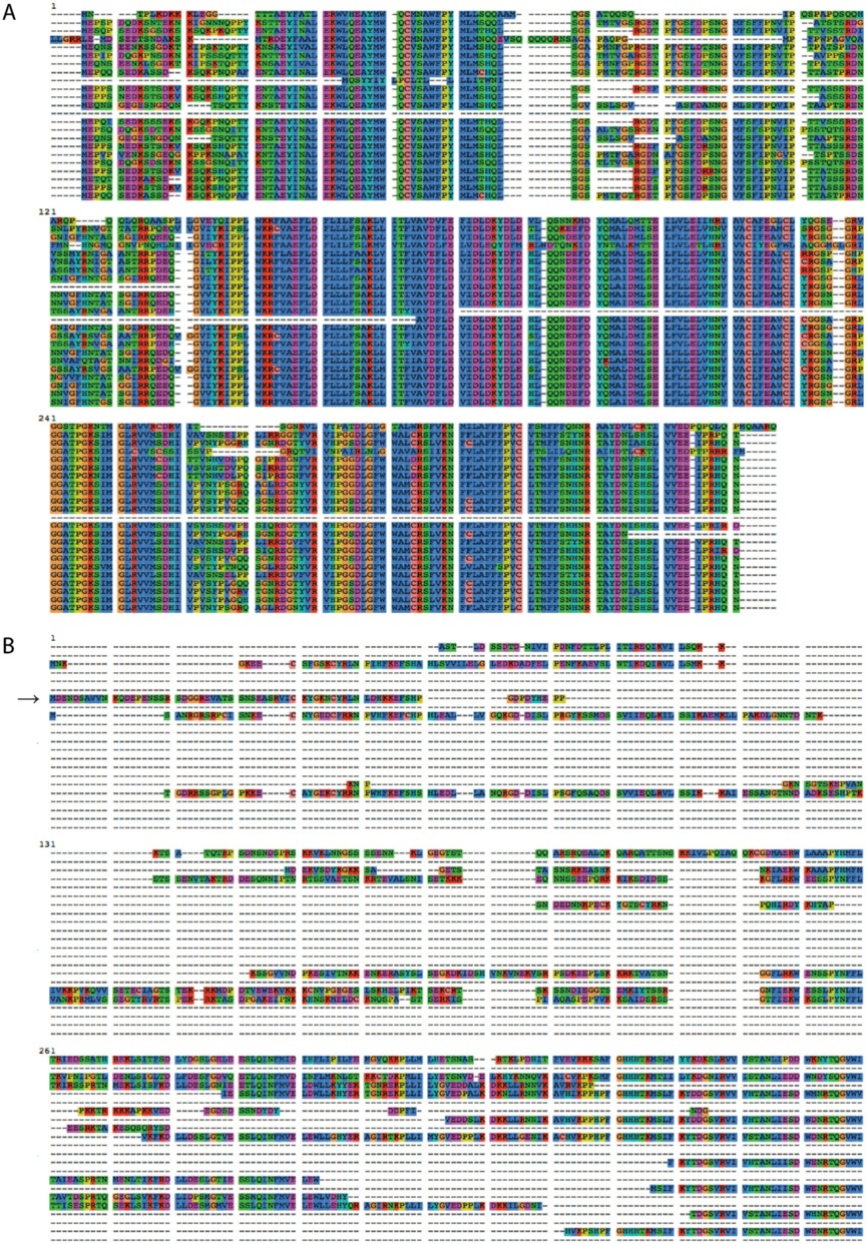
Examples of a high quality homology (**a**) and false-positive homology (**b**) clusters (OD_S data set) classified by meta-classifier w/ logistic regression. All sequences within the homology cluster (**a**) belong to one protein family (FAM81A1-like protein). The sequence in the false-positive homology cluster indicated by the arrow represents Aprataxin and PNK-like factor whereas other sequences represent tyrosyl-DNA phosphodiesterase

Lower coverage data sets are often used when performing transcriptomic and evolutionary analyses especially on non-model organisms. For instance, in a recent paper [32] the authors inferred a phylogeny of many insect species using relatively small RNA-seq library sizes averaging at ~ 3Gb (Additional file 2) compared to *Drosophila* data sets (Additional file 3). We expected the number of false positive clusters to increase with the decreasing sequencing depth. In order to examine this, three DROSO data sets were tested for the presence of false positives using the meta-classifier with logistic regression trained on the EQUAL arthropod data set. Indeed, we found that the number of false positive homology clusters increased in the subsampled DROSO data sets (15.7 %, 17.8 % and 29.9 % for 100 %, 50 % and 10 % DROSO data sets respectively). These subsampled data sets allowed us to see the results that are common when homology clustering is performed on small libraries. Applying the filtering process to the InParanoid and HaMStR OD_S clusters resulted in many removed clusters (Table 4), implying that heuristic-based methods have increased rates of false positives. For filtering, we only used the meta-classifier with logistic regression. The removal of many clusters showed the overall poor quality of many of the putative homology clusters (for comparison between homology and false-positive homology clusters see Fig. 4). This was expected due to the low quality transcriptome assembly that was caused by sequencing depth in addition to biological factors such as interspecific differential expression. The filtering process preserved higher quality clusters and finished almost instantly resulting in huge time savings when compared to manually curating the clusters. Overall our method can be applied to filter homology clusters derived from closely related (e.g. *Drosophila* species) as well as highly diverged taxa (e.g. Odonata species). We also note that the trimming procedure behaves more conservatively with increasingly diverged sequences.

**Table 4:** Summary of InParanoid and HaMStR cluster filtering

		Kept	Removed
Odonata	InParanoid	10500	3497
	HaMStR	1231	896

The number of clusters that were kept and removed for the OD_S clusters from InParanoid and HaMStR. Filtering was accomplished using the meta-classifier w/ logistic regression model trained on the EQUAL data set

## Conclusions

We have demonstrated a machine learning method that can be used to differentiate homology and non-homology clusters based on characteristics of known good and bad clusters. These results can be seen in our trained models’ ability to achieve high classification accuracy on the test data sets as well as by examining the number of clusters that were removed from the experimental OD_S data set. We developed a training set of known good and bad clusters that was previously unavailable and made supervised machine learning impossible. Using a feature set that we developed, we tested various machine learning algorithms and found that when trained on our training data sets that the meta-classifier with logistic regression consistently outperformed all other models and performed just as well as the meta-classifier without logistic regression.

Applications of our method were also seen as we applied them to other data sets. Our method was especially useful when applied to the OD_S data set, by filtering out many clusters with false positive homology. We showed that our method is effective in settings where non-model organisms are being studied and the transcriptome assembly quality is low primarily due to low coverage sequencing or partial RNA degradation.

This paper has demonstrated the usefulness of machine learning in finding homology clusters by quickly removing low quality clusters without using any additional heuristics. The clusters that are retained can then be used later in higher quality phylogeny reconstruction and/or other analyses of gene evolution. In the future, we aim to explore machine learning approaches to clustering sequences more deeply to produce more refined and reliable homology clusters.

## Additional files

Additional file 1:
**Summary of OD_S RNA-seq libraries.** (XLSX 44 kb) Additional file 2:
**Density estimation of RNA-seq base coverage used in [** 32 **].** (PDF 75 kb) Additional file 3:
**Summary of DROSO RNA-seq libraries.** (XLSX 40 kb)
