# Supplementary figures and images for: Detecting false positive sequence homology: a machine learning approach
Source: BMC Bioinformatics. 2016 Feb 24;17:101. doi: 10.1186/s12859-016-0955-3 (PMC4765110; doi:10.1186/s12859-016-0955-3)

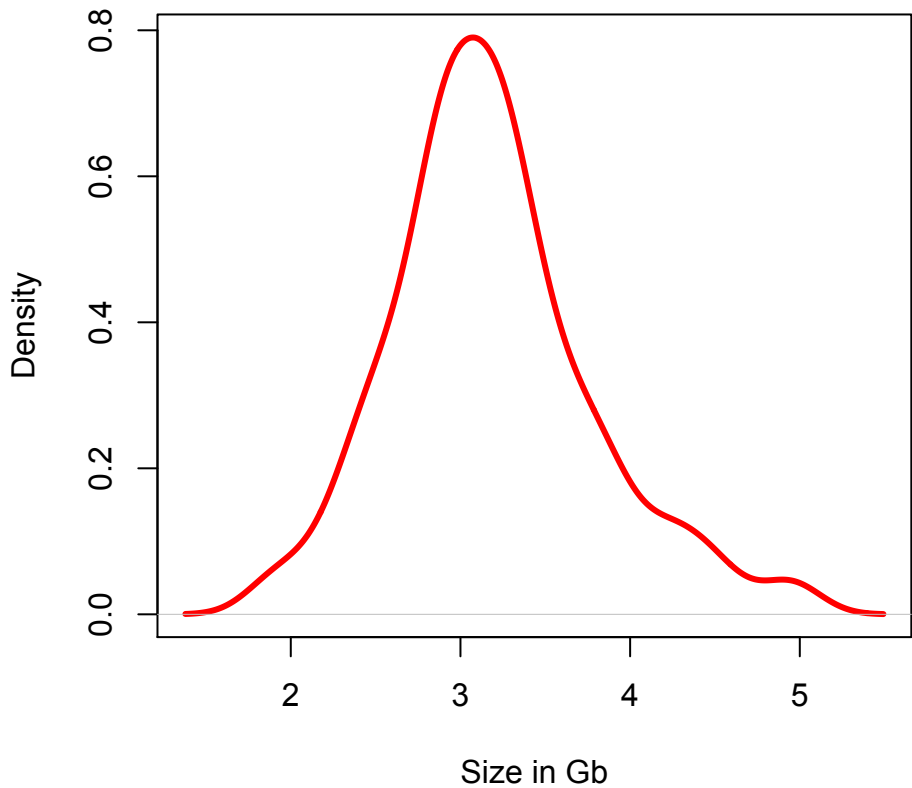

Supplement: Additional file 2: — Density estimation of RNA-seq base coverage used in [ 32 ]. (PDF 75 kb) [file 12859_2016_955_MOESM2_ESM.pdf]
